# In Vitro Transcriptional Response of *Eimeria tenella* to Toltrazuril Reveals That Oxidative Stress and Autophagy Contribute to Its Anticoccidial Effect

**DOI:** 10.3390/ijms24098370

**Published:** 2023-05-06

**Authors:** Lei Zhang, Hongtao Zhang, Shiqi Du, Xingju Song, Dandan Hu

**Affiliations:** 1 College of Animal Science and Technology, Guangxi University, Nanning 530004, China; 2 Guangxi Zhuang Autonomous Region Engineering Research Center of Veterinary Biologics, Nanning 530004, China; 3 Guangxi Key Laboratory of Animal Breeding, Disease Control and Prevention, Nanning 530004, China

**Keywords:** *Eimeria tenella*, toltrazuril, gene expression, ROS, RNA-seq

## Abstract

Intestinal coccidiosis is a common parasitic disease in livestock, caused by the infection of *Eimeria* and *Cystoisospora* parasites, which results in great economic losses to animal husbandry. Triazine compounds, such as toltrazuril and diclazuril, are widely used in the treatment and chemoprophylaxis of coccidiosis. Unfortunately, widespread drug resistance has compromised their effectiveness. Most studies have focused on prophylaxis and therapeutics with toltrazuril in flocks, while a comprehensive understanding of how toltrazuril treatment alters the transcriptome of *E. tenella* remains unknown. In this study, merozoites of *E. tenella* were treated in vitro with 0.5 μg/mL toltrazuril for 0, 1, 2 and 4 h, respectively. The gene transcription profiles were then compared by high-throughput sequencing. Our results showed that protein hydrolysis genes were significantly upregulated after drug treatment, while cell cycle-related genes were significantly downregulated, suggesting that toltrazuril may affect parasite division. The expression of redox-related genes was upregulated and elevated levels of ROS and autophagosomes were detected in the parasite after toltrazuril treatment, suggesting that toltrazuril may cause oxidative stress to parasite cells and lead to its autophagy. Our results provide basic knowledge of the response of *Eimeria* genes to toltrazuril and further analysis of the identified transcriptional changes can provide useful information for a better understanding of the mechanism of action of toltrazuril against *Eimeria*.

## 1. Introduction

Coccidian parasites (including *Eimeria* and *Cystoisospora* species) are known to infect the intestinal epithelial cells of farm animals, which leads to hematochezia, poor feed utilization ratio and mortality [1]. Coccidiosis is prevalent worldwide and causes significant losses in the farming industry, especially in the poultry industry [2,3,4]. Different strategies are currently used to control the disease, such as improving management and hygiene, using anticoccidial products or applying various types of anticoccidial vaccines [5,6,7]. However, the prophylactic application of coccidiostats in feed or drinking water remains the most widely distributed method for controlling coccidiosis worldwide [8]. In recent years, field resistance to coccidiostats has become increasingly common [9,10,11]. However, despite their widespread applications in farming, the mode of action of many coccidiostats remains unknown.

Toltrazuril, like diclazuril, is a triazine-based frontline anticoccidial drug that has been widely used in the field for decades [12]. The frequent use of toltrazuril has led to the development of resistance to *Eimeria* species in the field [10,13]. To better control coccidiosis, we need to investigate the mechanism of action of existing anticoccidial agents. Further understanding of its mechanism could reveal new targets for drug development against this disease.

Previous studies on the mode of action of toltrazuril in *Ascaris suum* have shown that the activities of certain enzymes in the respiratory chain (e.g., NADH oxidase and fumarate reductase) become reduced in the presence of toltrazuril [14]. In *Eimeria*, toltrazuril was found to act in all intracellular stages of either the schizogenic cycle or the gametogenic cycle. After treatment with toltrazuril, the perinuclear space, mitochondria and endoplasmic reticulum were found to be considerably enlarged in *E. tenella*; nuclear divisions were disturbed in schizonts and microgamonts [15]. Moreover, treatment of second-generation merozoites with diclazuril was found to induce morphological changes and attenuate the activity of the mitochondrial transmembrane potential of merozoites, which is associated with mitochondrial-dependent apoptosis [16]. Darius et al. [17] added different concentrations of toltrazuril to the cell culture infected with *Neospora caninum*, and then observed the proliferation and fine structure of tachyzoites (NC-1 strain) by optical and electron microscopy. It was found that toltrazuril led to a significant reduction in the number of tachyzoites in cultured cells and that morphological damage to the parasite increased with the increasing dosage of the drug, especially to the apicoplast and mitochondria. Bierbaum [18] used the phage display method to predict that the target of action of toltrazuril would be a 20.5 kDa cyclophilin, EtCyp20.5. Upon co-incubation of recombinant EtCyp20.5 with toltrazuril, the drug was found to inhibit the PPIase activity of this protein [18]. However, the comprehensive molecular mechanism underlying the effect of toltrazuril on *E. tenella* remains largely unknown.

Here, we report the early transcriptional profiles of *E. tenella* after treatment with toltrazuril in the hope that these results will contribute to the understanding of the mode of action of this coccidiostat and aid in the control of coccidiosis.

## 2. Results

### 2.1. Overview of Sequencing Data and Gene Expression Patterns

The merozoite stage causes the most lethal damage to the intestinal epithelial cells and overall physiological functions of the infected chicken [1]. Previous reports have shown that toltrazuril acts on the parasite during the intracellular stage [12]; as a result, in this study, second-generation merozoites were treated with toltrazuril in vitro. To gain insight into early gene expression and to avoid the noise from parasite death, merozoites were only treated for up to 4 h of time-course series and subsequently subjected to RNA-seq analysis.

**Figure 1:**
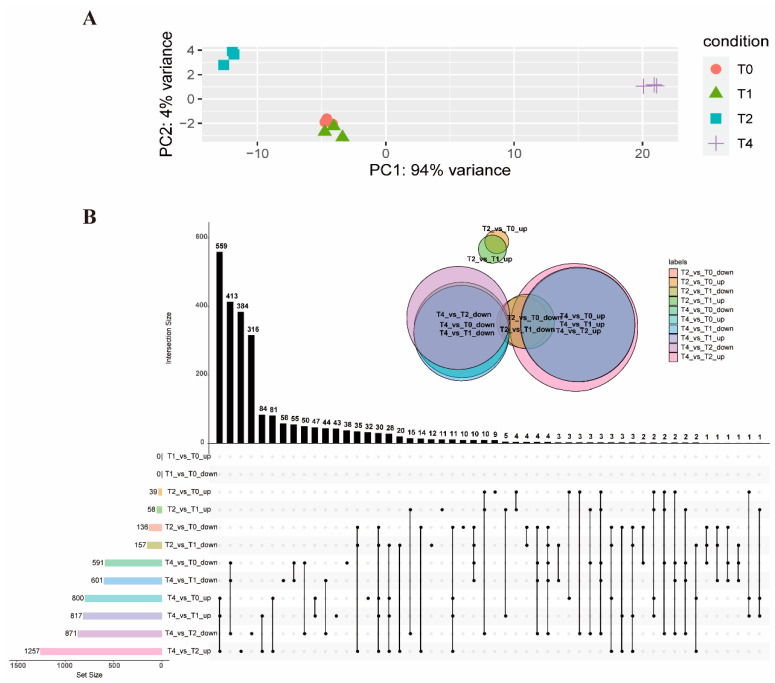
Overview of RNA-seq analysis. (**A**) PCA analysis of all RNA-seq samples. (**B**) An upset-plot showing sets of differentially expressed genes after toltrazuril treatment. Vertical bars show the number of intersecting genes between the comparison groups, denoted by the connected black dots below the histogram. Horizontal bars show the size of DEGs between the comparison groups. The Venn diagram shows the size and overlapping situation of DEGs in the different groups. Each black dot represents the differential genes of different comparison groups. If two black dots are connected in a straight line, the corresponding black histogram above represents the number of differential genes common to both comparison groups.

To gain a comprehensive understanding of the gene expression patterns in response to toltrazuril at different times, DEGs were identified between the different groups (Supplementary Dataset S1). The group treated with toltrazuril for 1 h (T1) did not show significantly different genes from the untreated group (T0), and they were characterized by highly similar information from a PCA analysis and Venn diagram (Figure 1A,B). Differentially expressed genes (DEGs) increased, along with the duration of toltrazuril exposure. There were 215 distinct genes differentially expressed between the 2 h (T2) and 1 h (Supplementary Dataset S2) treatments and 175 DEGs between the 2 h (T2) treatment and the untreated group (T0) (Supplementary Dataset S3, Figure S1). The highest number of DEGs (2128) was found in the comparison between 4 h (T4) and 2 h of toltrazuril treatment (Supplementary Dataset S4). There were 1418 DEGs between T4 and T1 (Supplementary Dataset S5) and 1391 DEGs between treatment at 4 h and T0 (Figure 1B, Supplementary Dataset S6, Figure S2).

### 2.2. Gene Alterations in E. tenella in Response to Toltrazuril Treatment

**Figure 2:**
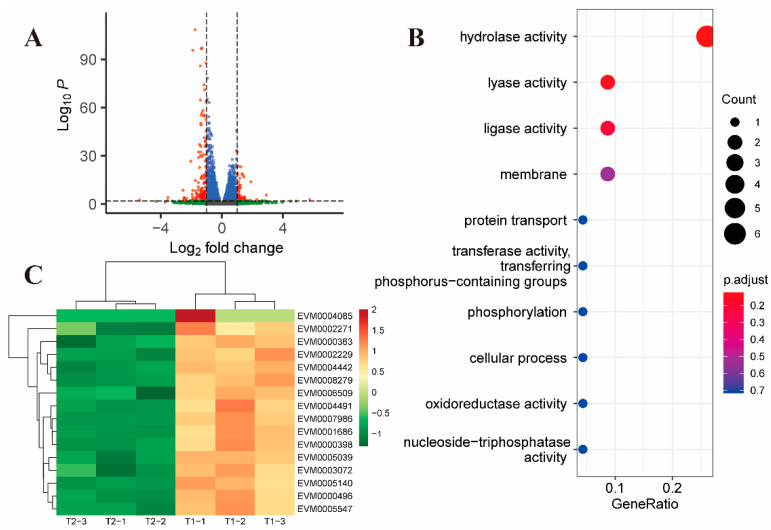
Differential gene expression after 2 h of toltrazuril treatment. (**A**) Volcano plot showing differentially expressed genes between T2 and T1 groups. Genes with an absolute fold change > 2 and an adjusted *p* value < 0.01 were considered significantly differentially expressed (red dots). (**B**) Gene ontology enrichment analysis of down-regulated differentially expressed genes between T2 and T1. The size of the bubble represents the number of genes and the color of the bubble is assigned based on the adjusted *p* value. (**C**) Heatmap showing differentially expressed genes associated with surface antigen proteins.

There were 215 differential genes after 2 h (T2) of treatment with toltrazuril compared to 1 h (T1), of which 58 genes were upregulated and 157 genes were downregulated (Figure 2A). GO enrichment analysis revealed that the down-regulated genes were mainly associated with hydrolase activity (GO:0016787, *p*-value = 0.0068, Figure 2B, Supplementary Dataset S7).

In addition to the down-regulation of surface antigen-associated genes (Figure 2C), two genes located in the inner mitochondrial membrane (EVM0004152 and EVM0003933) were found to be downregulated in the T2 and T1 comparison groups (Supplementary Dataset S2, Figure S3). EVM0004152, annotated as mitochondrial pyruvate carrier (MPC), is responsible for transporting pyruvate from cytosol to mitochondria in such a way that the inhibition of MPC activity would result in a significant decrease in glucose metabolism [19]. EVM0003933 is annotated as Prohibitin, an evolutionarily conserved and ubiquitously expressed mitochondrial protein. Studies have shown that Prohibitin deficiency in cells leads to increased production of reactive oxygen species (ROS) [20]; Matz JM et al. [21] found that the absence of Prohibitin (PHBL) leads to a decrease in the mitochondrial membrane potential in *Plasmodium*. However, this gene was differentially expressed only after 2 h of toltrazuril treatment, suggesting that it may play a transient role in the *Eimeria* parasite.

**Figure 3:**
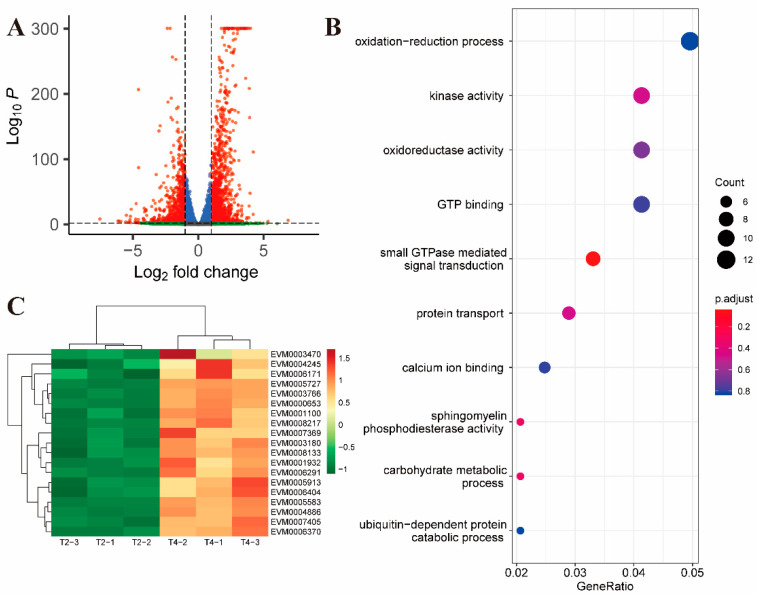
Differential gene expression between 2 and 4 h of toltrazuril treatment. (**A**) Volcano plot showing differentially expressed genes between T4 and T2 groups. Genes with an absolute fold change > 2 and an adjusted *p* value < 0.01 were considered significantly differentially expressed (red dots). (**B**) Gene ontology enrichment analysis of up-regulated differentially expressed genes between T4 and T2. The size of the bubble represents the number of genes and the color of the bubble is assigned based on the *p*-value. (**C**) Heatmap showing differentially expressed genes associated with vesicular transport and ubiquitination of proteins.

After 2 h of further exposure (compared to T4 and T2), there were 1257 up-regulated genes and 871 down-regulated genes (Figure 3A). Upregulated genes were enriched in the small GTPase-mediated signal transduction pathway (GO:0007264, *p* value = 0.0006) (Figure 3B, Supplementary Dataset S8). Unlike the T2 and T1 comparison groups, members of the SAG family were upregulated in the T4 and T2 comparison groups (Supplementary Dataset S3). In addition, most of the remaining up-regulated genes in the comparison between T4 and T2 were related to vesicular transport and ubiquitination of proteins (Figure 3C).

**Figure 4:**
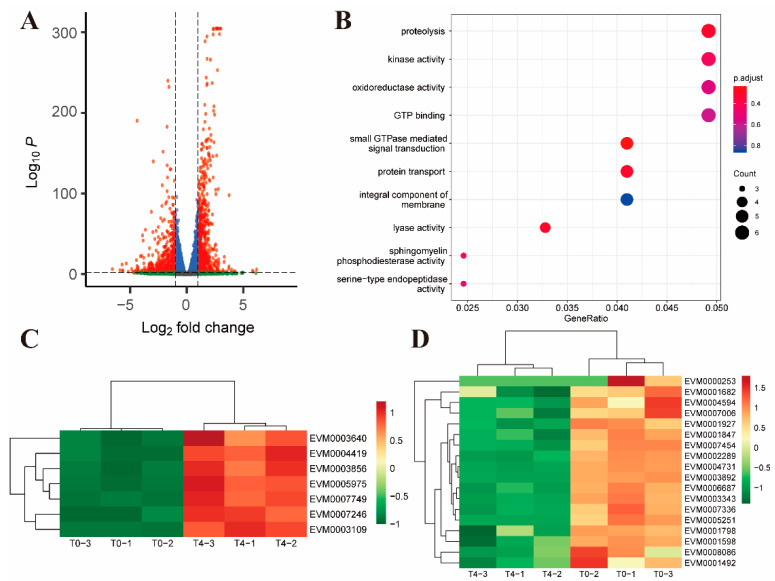
Differential gene expression between toltrazuril-treated (4 h) and untreated parasites. (**A**) Volcano plot showing the difference in gene expression between T4 and T0 groups. Genes with an absolute fold change > 2 and an adjusted *p* value < 0.01 were considered significantly differentially expressed (red dots). (**B**) Gene ontology enrichment analysis of up-regulated differentially expressed genes between the T4 and T0 groups. The size of the bubble represents the number of genes and the color of the bubble is assigned based on the adjusted *p* value. Clustered heatmaps showing differentially expressed genes associated with antioxidant proteins (**C**) and cell division-related proteins (**D**) between the T4 and T0 groups.

Compared to the untreated control, there were 1391 differential genes after 4 h of toltrazuril treatment, of which 800 genes were upregulated and 591 genes were downregulated (Figure 4A). Among the up-regulated genes, proteolysis-associated DEGs (GO: 0006508, *p* value = 0.021) were significantly enriched (Figure 4B, Supplementary Dataset S9). These results demonstrated an accelerated protein degradation process in parasites after 4 h of treatment. In addition, many surface antigens, inner membrane complex proteins and antioxidant proteins, such as thioredoxin and superoxide dismutase, were also upregulated (Figure 4C, Supplementary Dataset S5). However, many cell division-related proteins are downregulated, such as MOB kinase activator-like 1 (EVM0001798) [22,23] and NEK kinase (EVM0001492) [24] (Figure 4D). Mob1 protein has been shown to be important for both mitotic completion and cell plate formation in yeast [25]. In *Trypanosoma brucei*, RNA interference down-regulates MOB1 leading to delayed cytokinesis and possible cell cycle disorder by inhibiting the replication of kinetoplast [26]. Members of the Nek kinase family have been identified as cell cycle regulators throughout eukaryotes. The temperature-sensitive cell cycle-deficient strain VA15 of *T. gondii* (cell cycle arrest due to mitotic defects) can be rescued by complementation with Tgnek1, which indicates that TgNek1 is a key cell cycle regulator [27].

### 2.3. Toltrazuril Induces Intracellular Oxidative Stress and Autophagy in Parasites

**Figure 5:**
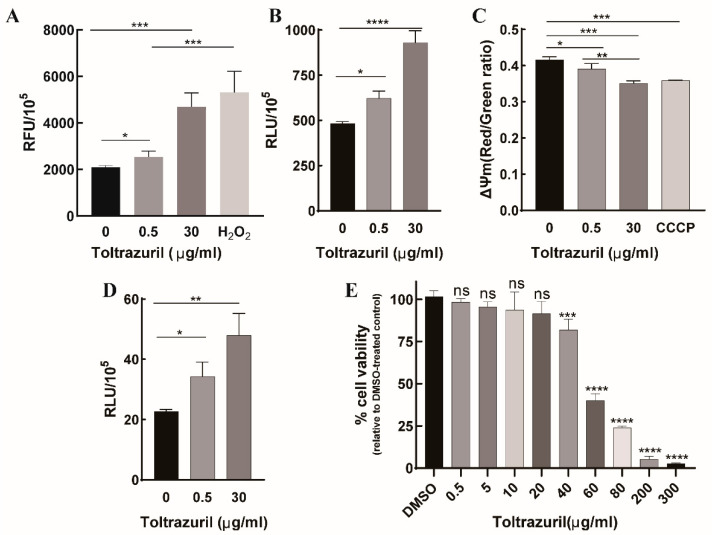
The effect of toltrazuril on merozoites and its cytotoxicity to DF1 cells. (**A**) Detection of ROS levels as relative fluorescence units per 1 × 10^5^ merozoites (RFU/10^5^) using DCFH-DA regent after incubation with different concentrations of toltrazuril for 4 h. (**B**) Detection of mitochondrial ROS levels as relative fluorescence units per 1 × 10^5^ merozoites (RFU/10^5^) using MitoSOX regent after incubation with different concentrations of toltrazuril for 4 h. (**C**) Detection of mitochondrial membrane potential by JC-10 MitoProbe after 4 h of treatment with toltrazuril. (**D**) Measurement of autophagosomes by MDC staining and detection of RFUs after incubation with different concentrations of toltrazuril for 4 h. (**E**) Cytotoxicity of toltrazuril on DF1 cells. DF1 cells were incubated with different concentrations of toltrazuril for 24 h and then cytotoxicity was determined using CCK-8 Reagent. The absorbance was measured at 450 nm. Data are expressed as mean ± SD in triplicate. Student’s *t*-test was used for statistical analysis. ns = not significant, and *p* values are represented by asterisks as follows: * *p* < 0.05, ** *p* < 0.01, *** *p* < 0.001 and **** *p* < 0.0001.

In the comparison between T4 and T0, we found that some up-regulated genes were associated with the oxidoreductive system, especially redox proteins. To verify whether up-regulated genes are related to oxidative stress, we detected ROS generation in parasite cells after incubation with 0.5 μg/mL and 30 μg/mL toltrazuril, respectively. Our results showed that ROS levels were significantly elevated after treatment with both concentrations of toltrazuril in a dose-dependent manner. Notably, the ROS level induced by 30 μg/mL toltrazuril was not significantly different from the H_2_O_2_ positive control (Figure 5A). Mitochondria is a major source of intracellular ROS in most cell types [28], so we used MitoSOX Red (MitoSOX) to detect mitochondrial ROS. The results showed that the pattern of mitochondrial ROS production was almost like that of intracellular ROS (Figure 5B). We also examined changes in mitochondrial membrane potential and found a decrease in membrane potential after drug treatment, with these results suggesting that toltrazuril may act on mitochondria (Figure 5C).

We also found that some autophagy-related genes, such as EVM0004969 (ATG18) [29] and EVM0003406 (ATG5) [30], were upregulated after 4 h of exposure to toltrazuril. This may be an indication that toltrazuril induces autophagy in parasites. To further test whether toltrazuril treatment induces autophagy, we detected the autophagosome of *E. tenella* merozoites after 4 h of exposure to toltrazuril by staining autophagic vacuoles with its specific marker MDC. MDC can emit green fluorescence after specifically labeling autophagosomes, and the fluorescence signals can be detected by a fluorescence spectrophotometer. After toltrazuril treatment, we found a significant increase in relative fluorescence intensities in both toltrazuril concentration groups, indicating that toltrazuril induces autophagy in merozoites (Figure 5D). Autophagy is an evolutionarily conserved cyclic process that responds to stress conditions, including the generation of a series of reactive oxygen species [31,32]. These results suggest that toltrazuril may lead to increased intracellular oxidative stress, which in turn causes parasite autophagy. In addition, for future drug development, the toxicity of toltrazuril on the chicken cell line DF1 was tested using CCK-8 Reagent, and the results showed that the proliferation of cells was significantly inhibited when the drug concentration reached 40 μg/mL (Figure 5E).

## 3. Discussion

Coccidiosis is one of the most important diseases affecting the farming industry [1]. According to the latest estimates, the global cost of coccidiosis to the poultry industry is about GBP 10 billion annually [33]. The control of coccidiosis relies mainly on prophylactic chemotherapy with anticoccidial drugs. Fully effective chemotherapeutic agents for the control of coccidiosis are challenging owing to the increasing problem of drug resistance [6]. Thus, understanding the mode of action of coccidiostats is essential for better utilization of these drugs and important for the development of new coccidiostats. As such, in this study, the early gene response of *E. tenella* merozoites to toltrazuril was dissected. Our data provide information on the early gene expression of *E. tenella* strains in response to toltrazuril treatment and provide important information for further studies on the mechanism of action of the triazine compounds.

Although the exact mechanism of action are presently not fully understood, triazines are considered to be anticoccidial compounds that may target both sexual and asexual life stages of protozoa. Triazines inhibit cytokinesis of merozoites and thus complete karyokinesis [12]. Lindsay et al. [34] found that treatment with diclazuril interfered with the endogenesis of *Toxoplasma gondii* (RH strain), resulting in the production of multinucleated (>2 nuclei) schizonts. After the treatment of *E. tenella*-infected chickens with diclazuril, first- and second-generation schizonts exhibited extensive degenerative changes [35]. It was observed via electron microscopy that the early growth and nuclear division proceeded normally in second-generation schizonts; however, the exogenesis of merozoites was largely prevented [36]. Nevertheless, the same changes were not observed in the other two species of chicken coccidia (*E. maxima* and *E. brunetti*) [37]. Mehlhorn et al. [15] found that toltrazuril significantly increased the perinuclear space, mitochondria and endoplasmic reticulum in *E. tenella*, *E. maxima* and *E. acervulina*. In this study, we also observed that several cell cycle-related genes were downregulated, while inner membrane complex-related genes were upregulated after exposure to toltrazuril for 4 h. These results also confirm that triazine inhibits the cell cycle of merozoites, which in turn inhibits their division.

Studies have shown that triazine compounds can cause mitochondrial dysfunction. Zhou et al. [16] found that diclazuril treatment led to a decrease in mitochondrial membrane potential, which could possibly further induce apoptosis. After treatment with toltrazuril, the apicoplast of *N. caninum* was destroyed, and focal swelling of tubular mitochondria and endoplasmic reticulum occurred [17]. The activities of NADH oxidase and fumarate reductase were found to be inhibited when *A. suum* was treated with toltrazuril. In our study, several genes related to mitochondria were upregulated after 4 h of treatment. These genes are mitochondrial carrier proteins and enzymes related to energy metabolism, such as cytochrome c oxidase subunit 1 and acyl CoA dehydrogenase. Down-regulated mitochondrial ribosomal proteins and some transposable enzymes, such as TOM40 [38], TOM22 [39] and PAM16 [40], may affect the entry of nuclear-encoded proteins into the mitochondria as well as proteins encoded by the mitochondria themselves. More importantly, we also found a decrease in the mitochondrial membrane potential of merozoites after treatment with toltrazuril and, taken together, these results suggest that toltrazuril treatment leads to mitochondrial dysfunction of the parasite.

After 2 h of treatment with toltrazuril, down-regulated DEGs were enriched in the molecular function category, including hydrolase activity, lyase activity and ligase activity, respectively. EVM0003933 was annotated as Prohibitin, an evolutionarily conserved and universally expressed mitochondrial membrane protein. Matz et al. [21] found that deletion of a prohibited protein (PHBL) resulted in a reduced mitochondrial membrane potential in malaria parasites. However, this gene was differentially expressed only after 2 h of treatment with toltrazuril, suggesting that it may play a temporary role in the *Eimeria* parasites.

After 4 h of treatment with toltrazuril, most of the up-regulated differential genes were enriched in proteolysis and oxidoreductase activity. This suggests that drugs may cause hydrolysis of parasite proteins and oxidative stress reactions in the parasite. Superoxide dismutase [41] (EVM0004419) and glutathione reductase [42] (EVM0003109) were involved in controlling the redox status of apicomplexan parasites [43], and the expression of these two genes was upregulated after 4 h of toltrazuril treatment, suggesting a possible resistance of the parasite to drug-induced redox changes at some degree for a short period of time.

In addition, we found that the expression level of enolase decreased after the action of toltrazuril. In addition to its innate glycolytic function, enolase has a variety of functions and may also be involved in the invasion process of parasites and the control of gene regulation [44]. Both diclazuril and acetamizuril have been reported to reduce the expression of enolase on the second generation merozoite, and it is speculated that enolase may be the target of the action of these two drugs [45,46]. Thus, we elucidated that toltrazuril exerts anticoccidial activity by interfering with merozoite metabolism. We also found that several antioxidant genes, such as thioredoxin, were upregulated after exposure to toltrazuril for 4 h, possibly indicating that toltrazuril induces oxidative stress in parasites, which was further verified by ROS measurements. We also demonstrated that autophagosomes of merozoites were elevated by toltrazuril via the MDC method and were accompanied by a significant upregulation of the small GTPase RAB protein family (Supplementary Dataset S6), such as Rab5 and Rab7, which play a role in the early and late stages of autophagy formation, respectively [47,48]. This probably indicates that the cell death process begins in the parasite. Previous studies have shown that oxidative stress also leads to the generation of autophagic responses [49,50,51]. Therefore, we speculate that toltrazuril may lead to parasite autophagy by enhancing oxidative stress.

## 4. Materials and Methods

### 4.1. Animals and Parasites

The *E. tenella* Houghton (ETH) strain was used throughout this work. The parasites were maintained and propagated by oral infection in one-week-old broilers (Sanhuang chicken, Fufeng Animal Husbandry Co., Ltd., Nanning, China). Four-week-old broilers were used to prepare merozoites. Chickens were reared in a coccidia-free environment. Filtered water and feed free of anticoccidials and antibiotics were provided ad libitum. Procedures for parasite collection, purification and sporulation were previously described [52].

### 4.2. Compounds

Toltrazuril (BAY-i9142, CAS#69004-03-1, ACS grade) was purchased from MedChemExpress (Shanghai, China). A stock solution (0.5 mg/mL) of toltrazuril was solubilized in dimethyl sulfoxide (DMSO), aliquoted and stored at −20 °C until use.

### 4.3. Merozoites Purification and Toltrazuril Treatment

Merozoites were collected as reported by Schwarz et al. [53] with modifications. Briefly, one-month-old broilers were randomly divided into three groups of three birds per group. Each chicken was inoculated orally with 5 × 10^5^ sporulated oocysts and then killed at 120 h post-infection to obtain the cecum. The sheared cecum was digested at 42 °C for 30 min with 0.50% sodium taurodeoxycholate hydrate and 0.25% trypsin in PBS solution, then filtered through gauze and centrifuged to obtain sediment containing dirty merozoites, which were further filtered to obtain clean merozoites. In contrast to previous studies, we treated the merozoites with toltrazuril in vitro. Before and after in vitro culture, the survival rate of merozoites was detected by trypan blue staining. Only merozoites with a >95% survival rate were used. For toltrazuril treatment, about 1.0 × 10^7^ fresh merozoites were cultured in DMEM containing 0.5 µg/mL Toltrazuril for 0 (T0), 1 (T1), 2 (T2) and 4 h (T4), respectively. All samples were resuspended with TRIzol reagent (Invitrogen, Beijing, China) after being washed twice with ice-cold PBS and then immediately stored at −80 °C. Each treatment consisted of three biological replicates.

### 4.4. RNA Extraction, Library Preparation and RNA-Seq

Total RNA was isolated by TRIzol reagent and genomic DNA was removed by DNase I (Tiangen Biotech Co., Ltd., Beijing, China). NanoPhotometer^®^ (IMPLEN, Los Angeles County, CA, USA), Qubit^®^ RNA Assay Kit on Qubit^®^ 2.0 Fluorometer (Life Technologies, Carlsbad, CA, USA) and RNA Nano 6000 Assay Kit on Bioanalyzer 2100 system (Agilent Technologies, Santa Clara, CA, USA) were used to test RNA purity, concentration and integrity, respectively. Only qualified samples were used for library preparation. Sequencing libraries were generated using the TruseqTM RNA Sample Prep Kit (Illumina, San Diego, CA, USA) as recommended by the manufacturer of the instrument. Sequencing was performed using the Illumina NovaSeq 6000 platform to produce 150 bp paired-end reads. The original sequencing data can be found in the Sequence Read Archive database under accession number PRJNA903782.

### 4.5. Bioinformatics

Using Hisat2 version 2.2.1 [54], pair-end clean reads were aligned to our newly generated reference genome of *E. tenella* H strain (chromosome-level genome deposited in the CNGB Sequence Archive of China National GeneBank DataBase under accession number CNP0003153). After converting the output SAM file to a BAM file, it was then sorted and indexed. The resulting BAM files were subsequently used for read count via htseq-count version 0.13.5 [55]. Differentially expressed genes between groups were calculated by the R package DEseq2 [56] and functional enrichment analysis (GO and KEGG) was performed using ClusterProfiler (v 4.0.5) [57]. Gene expression with a fold change > 2 or <−2 and an adjusted *p* value < 0.01 was considered significantly differentially expressed. Transcripts per million (TPM) were calculated for each gene and used for clustered heatmap drawing.

### 4.6. Real-Time PCR

To verify the gene expression data, six DEGs were selected for qPCR validation. Total RNA (1 μg) was reverse-transcribed into cDNA using EasyScript^®^ First-Strand cDNA Synthesis SuperMix (TransGen Biotech, Beijing, China). The real-time PCR reaction was performed using the TransScript^®^ II Green One-Step qRT-PCR SuperMix (TransGen Biotech, Beijing, China) and the Roche LightCycler^®^ 480 system. Primers are described in Table S1. The expression of each gene was normalized to the reference gene glyceraldehyde 3-phosphate dehydrogenase (GAPDH) [58].

### 4.7. Measurement of Intracellular ROS Levels

Intracellular ROS levels were measured using the Reactive Oxygen Species Assay Kit (Beyotime Biotechnology, Shanghai, China) following the manufacturer’s instructions. DCFH-DA (2′,7′-dichlorofluorescein-diacetate) can be easily oxidized to fluorescent dichlorofluorescein (DCF) by intracellular ROS, and ROS levels can then be quantified by the fluorescent signals. Briefly, merozoites were purified as described above and exposed to various concentrations of toltrazuril for 4 h. After treatment, merozoites were incubated with DCFH-DA for 20 min at 37 °C and then the fluorescent signals were measured at 488 nm excitation and 525 nm emission using a fluorescence spectrophotometer (Tecan, Infinite M200 PRO, Männedorf, Switzerland). H_2_O_2_ (200 µM) served as a positive control.

### 4.8. Mitochondrial ROS (mROS) Measurement

mROS was measured using MitoSOX Red (MedChemExpress, Shanghai, China) according to the manufacturer’s instructions. Briefly, after incubation of merozoites with different concentrations of drugs, merozoites from all groups were incubated with 5 μM MitoSOX for 15 min at 37 °C in the dark. After washing 3 times with warm DMEM, we detected the ROS intensity using a fluorescence spectrophotometer (Tecan, Infinite M200 PRO, Männedorf, Switzerland).

### 4.9. Measurement of Mitochondrial Membrane Potential

The mitochondrial membrane potential was monitored using the JC-10 assay kit (Beijing 4A Biotech Co., Ltd., Beijing, China). Briefly, drug-treated merozoites were incubated with 10 μM JC-10 dye solution at 37 °C (protected from light) for 30 min and then washed three times with DMEM before measuring the red and green fluorescence intensities using a fluorescence spectrophotometer. Concentration with a lipophilic dye resulted in the formation of red fluorescent J-aggregates in healthy mitochondria and green fluorescent j-monomers in damaged mitochondria. The mitochondrial membrane potential was quantified as red fluorescence intensity/green fluorescence intensity. A decrease in the red signal indicates a decrease in the mitochondrial membrane potential. CCCP (carbonyl cyanide 3-chlorophenylhydrazone, 10 μM) served as a positive control to induce a decrease in mitochondrial membrane potential.

### 4.10. Autophagy Detection

Parasite autophagy was measured by using Monodansylcadaverine (MDC, Beyotime Biotechnology, Shanghai, China) as previously reported. MDC as a specific marker of autophagic vacuoles is an auto-fluorescent compound due to the conjugation of dansyl with cadaverine, a diaminepentan. MDC can be used as a good staining marker to evaluate the formation of autophagosomes. After treatment, *E. tenella* merozoites were incubated with MDC stain for 30 min at 37 °C, followed by fluorescence measurements at 335 nm excitation and 512 nm emissions using a fluorescence spectrophotometer (Tecan, Infinite M200 PRO, Männedorf, Switzerland).

### 4.11. Cytotoxicity Assay

The cytotoxicity of toltrazuril was evaluated in the DF1 cell line using CCK-8 reagents. Briefly, DF1 cells (4000 cells/well) were cultured in 96-well plates at 37 °C and 5% CO_2_ for 24 h. Cells were then treated with different concentrations of toltrazuril (0.5–300 μg/mL) for 24 h before determining cytotoxicity using CCK-8 reagent according to the manufacturer’s instructions (Beyotime, Shanghai, China). DMSO was added as a control. Absorbance was measured at 450 nm using a microplate absorbance reader (BioRad, Hercules, CA, USA). Data were obtained from three independent experiments.

## 5. Conclusions

In this study, the early gene response of *E. tenella* merozoites to toltrazuril was analyzed by comparative transcriptome. Cell cycle-related genes were significantly downregulated in toltrazuril-treated merozoites, suggesting that toltrazuril may affect merozoite division. Moreover, we demonstrated that toltrazuril decreased the mitochondrial membrane potential of merozoites and induced autophagy production by inducing ROS production. Our data provide valuable information for further investigation of the genetic mechanism of action of anti-coccidia triazine compounds.

## Data Availability

The datasets generated during this study have been uploaded to NCBI (No. PRJNA903782).

## Supplementary Materials

The following supporting information can be downloaded at: https://www.mdpi.com/article/10.3390/ijms24098370/s1.
