# Supplementary material for: In Vitro Transcriptional Response of Eimeria tenella to Toltrazuril Reveals That Oxidative Stress and Autophagy Contribute to Its Anticoccidial Effect
Source: Int J Mol Sci. 2023 May 6;24(9):8370. doi: 10.3390/ijms24098370 (PMC10179680; doi:10.3390/ijms24098370)
Supplement: Supplementary file 1 [file ijms-24-08370-s001.zip › Supplementary Figures.pdf]

Supplementary Figure S1. Heatmap showing differentially expressed genes between 2 h of toltrazuril treatment and no treatment (T2 vs. T0).

Supplementary Figure S2. Heatmap showing differentially expressed genes between 4 h of toltrazuril treatment and no treatment (T4 vs. T0).

Supplementary Figure S3. qPCR validation of differentially expressed genes. Statistical analysis was performed using unpaired two-tailed Student's *t*-tests with glyceraldehyde-3-phosphate dehydrogenase (GAPDH) as the reference.
