# Supplementary material for: In Vitro Transcriptional Response of Eimeria tenella to Toltrazuril Reveals That Oxidative Stress and Autophagy Contribute to Its Anticoccidial Effect
Source: Int J Mol Sci. 2023 May 6;24(9):8370. doi: 10.3390/ijms24098370 (PMC10179680; doi:10.3390/ijms24098370)
Supplement: Supplementary file 1 [file ijms-24-08370-s001.zip › supplementary Table S1.pdf]

Supplementary Table S1： Primers used for validation of the presence of differentially expressed gene (DEG) transcripts using Real time-PCR.

Primers	5’-3’
EVM0004152-F	CGCACCTACGGACGCACAAC
EVM0004152-R	CATAAGACCCCAGTCATTCGCTCAC
EVM0006509-F	TCCAGCACCAGAGGAAGGAGAAC
EVM0006509-R	CGGCAGTGAGGGACTTGAAAGC
EVM0004969-F	CCTTGCTATGGTGACGGACTTCG
EVM0004969-R	CCGCTCGTTGTTCCCTGTGTG
EVM0003960-F	TAGGAGCTTCTGAGGTAGCTTTCCC
EVM0003960-R	TCCAACCAACACCTGAACCAAACTC
EVM0007006-F	GGACAAGAGCAGCAGACAGGAAC
EVM0007006-R	CCGCCTCGAATTTGCCCTCAG
EVM0001682-F	GCAGCAGCAGGAAGCAGATGAG
EVM0001682-R	TTGGCAGCACTGAGGTTGTTGG
GAPDH-F	CATTGGGCGGTTGGTCTTCC
GAPDH-R	CCAGGATATCTGCCGTGGAC
