# Supplementary figures and images for: In Vitro Transcriptional Response of Eimeria tenella to Toltrazuril Reveals That Oxidative Stress and Autophagy Contribute to Its Anticoccidial Effect
Source: Int J Mol Sci. 2023 May 6;24(9):8370. doi: 10.3390/ijms24098370 (PMC10179680; doi:10.3390/ijms24098370)

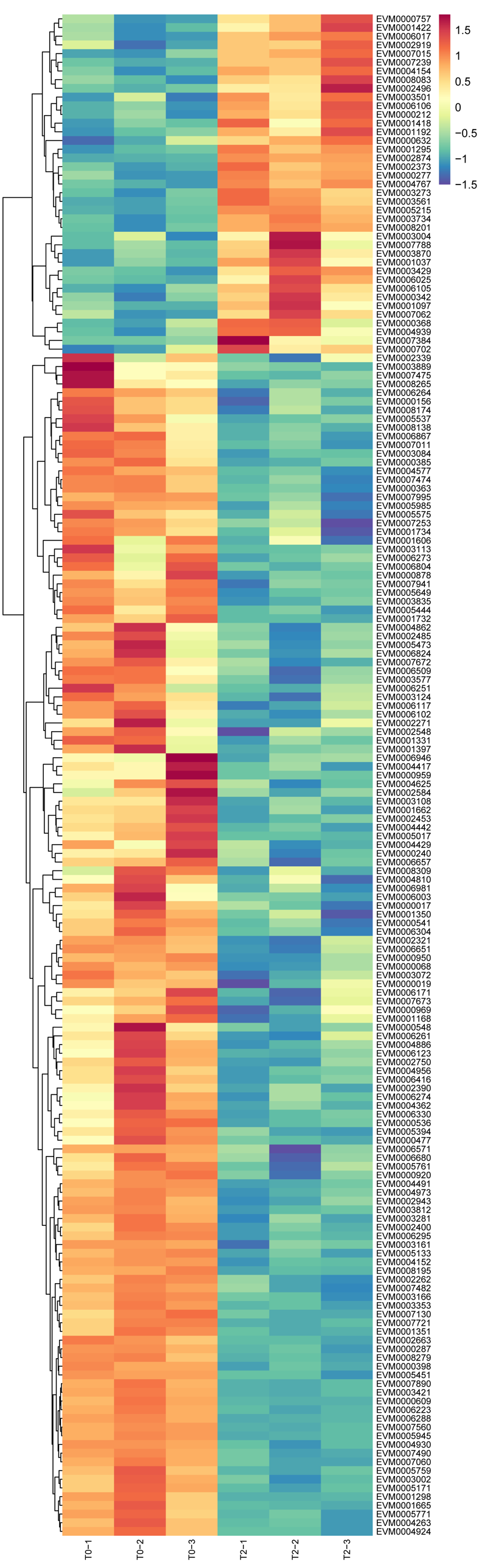

Supplement: Supplementary file 1 [file ijms-24-08370-s001.zip › Supplementary Figure S1.tif]

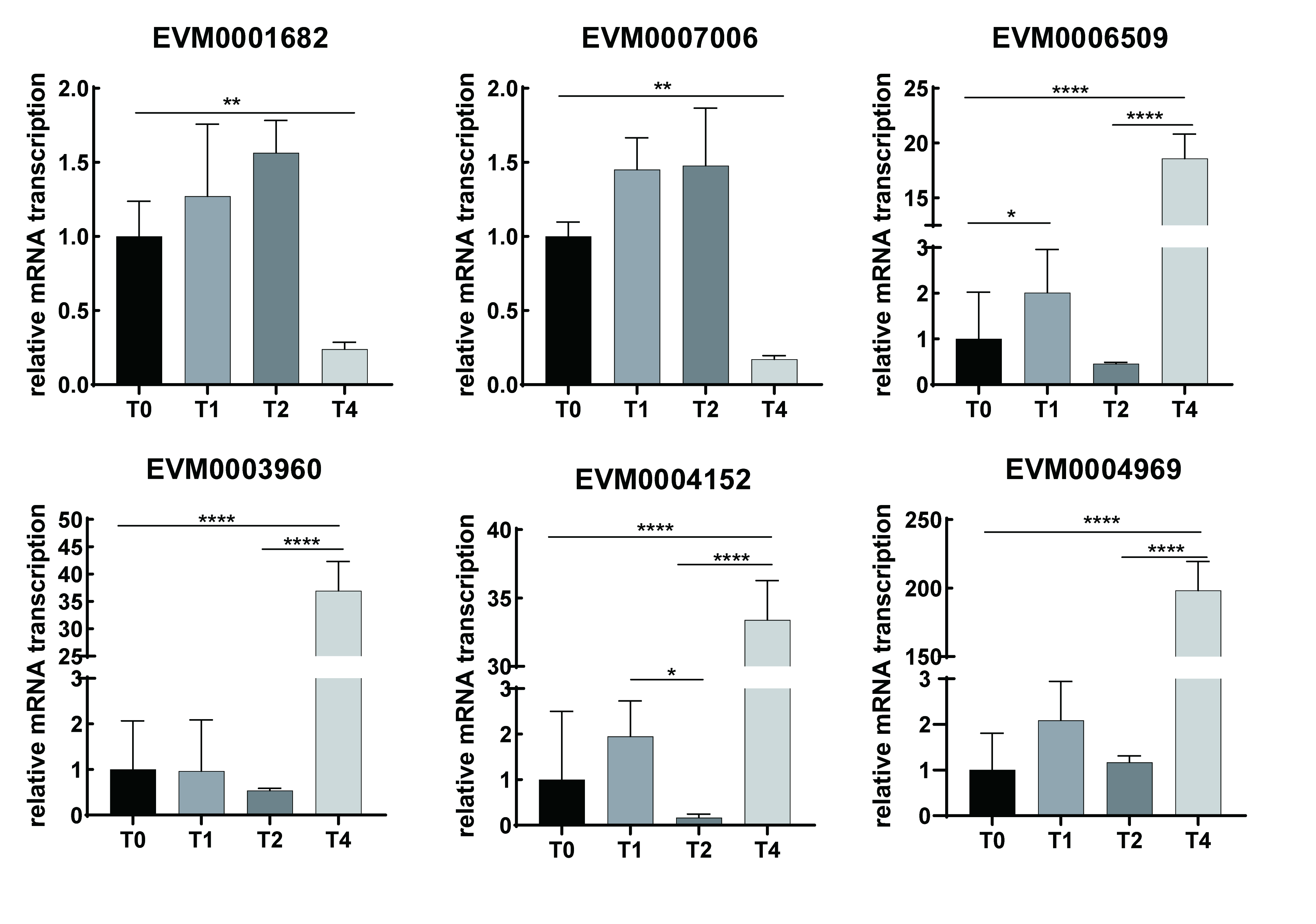

Supplement: Supplementary file 1 [file ijms-24-08370-s001.zip › Supplementary figure S3.tif]
